# Incidence Rate of Needlestick and Sharps Injuries in 67 Japanese Hospitals: A National Surveillance Study

**DOI:** 10.1371/journal.pone.0077524

**Published:** 2013-10-30

**Authors:** Toru Yoshikawa, Koji Wada, Jong Ja Lee, Toshihiro Mitsuda, Kiyoshi Kidouchi, Hitomi Kurosu, Yuji Morisawa, Mayumi Aminaka, Takashi Okubo, Satoshi Kimura, Kyoji Moriya

**Affiliations:** 1 Department of Research, The Institute for Science of Labour, Kawasaki, Japan; 2 Bureau of International Medical Cooperation, National Center for Global Health and Medicine, Tokyo, Japan; 3 Department of Infection Control and Prevention, Kobe University Hospital, Kobe, Japan; 4 Department of Infection Prevention and Control, Yokohama City University Hospital, Yokohama, Japan; 5 Tono Public Health Center, Gifu, Japan; 6 Department of Nursing, Tokyo Metropolitan Health and Medical Treatment Corporation Ebara Hospital, Tokyo, Japan; 7 Department of Infection Prevention and Control, Jichi Medical University Hospital, Tochigi, Japan; 8 National college of Nursing, Tokyo, Japan; 9 Department of Infection Prevention and Control, Tokyo Healthcare University Postgraduate School, Tokyo, Japan; 10 Tokyo Healthcare University, Tokyo, Japan; 11 Department of Infection control and prevention, The University of Tokyo, Tokyo, Japan; National Institute of Allergy and Infectious Diseases, United States of America

## Abstract

**Background:**

Determining incidence rates of needlestick and sharps injuries (NSIs) using data from multiple hospitals may help hospitals to compare their in-house data with national averages and thereby institute relevant measures to minimize NSIs. We aimed to determine the incidence rate of NSIs using the nationwide EPINet surveillance system.

**Methodology/Principal Findings:**

Data were analyzed from 5,463 cases collected between April 2009 and March 2011 from 67 Japanese HIV/AIDS referral hospitals that participated in EPINet-Japan. The NSI incidence rate was calculated as the annual number of cases with NSIs per 100 occupied beds, according to the demographic characteristics of the injured person, place, timing, device, and the patients’ infectious status. The NSI incidence rates according to hospital size were analyzed by a non-parametric test of trend. The mean number of cases with NSIs per 100 occupied beds per year was 4.8 (95% confidence interval, 4.1–5.6) for 25 hospitals with 399 or fewer beds, 6.7 (5.9–7.4) for 24 hospitals with 400–799 beds, and 7.6 (6.7–8.5) for 18 hospitals with 800 or more beds (p-trend<0.01). NSIs frequently occurred in health care workers in their 20 s; the NSI incidence rate for this age group was 2.1 (1.6–2.5) for hospitals having 399 or fewer beds, 3.5 (3.0–4.1) for hospitals with 400–799 beds, and 4.5 (3.9–5.0) for hospitals with 800 or more beds (p-trend<0.01).

**Conclusions/Significance:**

The incidence rate of NSIs tended to be higher for larger hospitals and in workers aged less than 40 years; injury occurrence was more likely to occur in places such as patient rooms and operating rooms. Application of the NSI incidence rates by hospital size, as a benchmark, could allow individual hospitals to compare their NSI incidence rates with those of other institutions, which could facilitate the development of adequate control strategies.

## Introduction

Needlestick and sharps injuries (NSIs) are major risks for blood-borne pathogen exposure in hospitals [1], [2]. The risk associated with NSIs varies depending on the devices, sharps waste management practices, degree of experience of health care workers, training opportunities, and the level of universal precaution practices [2], [3]. Various measures have been implemented in the United States to minimize the risk of sustaining NSIs, such as the proper use of safety-engineered devices, and the regulatory requirement that these devices be provided; these have reduced the incidence of NSIs [4], [5].

Hospitals may monitor NSIs by using adequate surveillance methods, for example EPINet, a tool used in the United States and other countries for monitoring NSIs, in order to develop a strategy for minimizing NSIs [4], [6]. EPINet was developed by the International Healthcare Worker Safety Center at the University of Virginia in 1992 [7]. In Japan, nationwide EPINet surveillance projects have used it since 1996 [8], [9].

A benchmark for the incidence rate of NSIs would allow hospitals to compare their data with those of other institutions and also assist with the development of measures to minimize NSIs [4], [5], [10]. There have been few studies focusing on the incidence rate of NSIs across a range of hospitals in Japan [11], [12], although some have examined rates in individual hospitals [13]–[16]. The aim of this study was to determine the incidence rate of NSIs in 67 hospitals located throughout Japan using the nationwide EPINet surveillance system.

## Materials and Methods

### Ethics Statement

The Human Research Committee at the Institute for Science of Labour approved the research methods and processes prior to study commencement (No. 2009-01).

### Data Collection

We targeted the HIV/AIDS referral hospitals in Japan since these hospitals are designated as secondary or tertiary care hospitals in their regions and are distributed geographically throughout Japan. These hospitals are also expected to have better precautions for needle stick injuries in place [17]. In 2008, participation agreement forms were sent to the directors of all 364 HIV/AIDS referral hospitals in Japan. Agreement to voluntarily participate in the study was obtained from 117 institutions. The infection control team at each hospital required all workers to report any NSIs and record each case using the EPINet-Japan form. In July 2011, we asked all 117 institutions to provide individualized NSIs data that had occurred between April 2009 and March 2011. We received individualized data from 67 of the 117 institutions.

### Statistical Analysis

Participating institutions were asked to report the number of approved beds by their local government and the mean annual bed occupation rate to calculate the actual number of occupied beds. The number of NSIs per year at each hospital was divided by the corresponding number of occupied beds, and the mean NSI incidence rate per 100 occupied beds and 95% confidence interval (CI) were calculated. The incidence rates were also classified according to the characteristics of the injured persons (e.g. age and job title), place (e.g. operating room), timing (e.g. when recapping the needle), and the devices involved.

The participating hospitals were then classified into three groups according to the number of occupied beds, i.e. those with 399 or fewer beds, those with 400–799 beds, and those with 800 or more beds. This was because of the variance between hospitals of different sizes with regard to their utilization of procedures associated with a high NSI risk, number of patients, number of health care workers, and the infection control system measures used. The NSI incidence rates of each category according to hospital size were analyzed by a non-parametric test of trend to identify any trends among the three hospital sizes. We analyzed the data using Stata version 11 (Stata Corp, College Station, TX).

## Results

The distribution of the hospitals that participated by location (n = 67) was evenly distributed throughout the country. The number of hospitals was highest in the Chubu area. The number of hospitals with a daily average of 399 or fewer occupied beds, 400–799 beds, or 800 or more beds, was 25, 24 and 18 hospitals, respectively.

The total number of reported NSI incidents was 2,680 from April 2009 to March 2010, and 2,783 from April 2010 to March 2011, equating to 5,463 incidents for the study period. Table 1 shows the annual NSI incidence rate per 100 occupied beds in relation to the demographic characteristics of the injured persons, place, timing, device, and the patients’ infectious status. The mean annual NSI incidence rate per 100 occupied beds with 95% CI was 6.2 (5.7–6.7). The injured health care workers were most frequently aged in their 20 s (3.2 [2.9–3.6]); nursing was the most frequently reported occupation (3.2 [3.0–3.5]). NSIs occurred predominantly in patient rooms (2.0 [1.8–2.2]) and operating rooms (1.7 [1.5–1.9]). NSIs most often occurred during the use of medical devices (1.7 [1.5–1.8]), and involved mainly disposable syringes (1.6 [1.4–1.7]) and suture needles (1.0 [0.9–1.2]).

**Table 1:** Number of needle stick injuries and mean incidence rate per 100 beds per year (n = 67 hospitals).

	N	(%)	Mean	(95%CI)
Total	5,463		6.2	(5.7–6.7)
Ages of injured health care workers (years)				
20–29	2,946	(53.9)	3.2	(2.9–3.6)
30–39	1,602	(29.3)	1.9	(1.7–2.1)
40–49	583	(10.7)	0.7	(0.7–0.8)
50+	332	(6.1)	0.4	(0.3–0.4)
Occupation of injured health care workers				
Doctors	1,882	(34.4)	2.1	(1.9–2.4)
Nurses	2,838	(51.9)	3.2	(3.0–3.5)
Place injuries occurred				
Patient room	1,728	(31.6)	2.0	(1.8–2.2)
Operating room	1,451	(26.6)	1.7	(1.5–1.9)
Outside of patient room	546	(10.0)	0.6	(0.5–0.7)
Outpatient clinic/office	473	(8.7)	0.5	(0.5–0.6)
Intensive/critical care unit	214	(3.9)	0.2	(0.2–0.3)
Emergency department	207	(3.8)	0.2	(0.2–0.3)
Timing of injury during device use				
Before use of device	246	(4.5)	0.3	(0.2–0.3)
During use of device	1,431	(26.2)	1.7	(1.5–1.8)
Between steps of a multi-step procedure	592	(10.8)	0.7	(0.6–0.8)
Disassembling device or equipment	402	(7.4)	0.4	(0.4–0.5)
In preparation for reuse of a reusable instrument	136	(2.5)	0.2	(0.1–0.2)
While recapping a used needle	476	(8.7)	0.6	(0.5–0.7)
Withdrawing a needle from rubber or another resistant material	170	(3.1)	0.2	(0.1–0.2)
Other after use-before disposal	479	(8.8)	0.5	(0.4–0.6)
While putting the device into a disposal container	409	(7.5)	0.5	(0.4–0.5)
Restraining patient	194	(3.6)	0.2	(0.2–0.3)
Devices causing injuries				
Disposable syringe	1,388	(25.4)	1.6	(1.4–1.7)
Suture needle	910	(16.7)	1.0	(0.9–1.2)
Winged steel needle	629	(11.5)	0.7	(0.6–0.8)
Pre-filled cartridge syringe	424	(7.8)	0.5	(0.4–0.6)
IV catheter stylet	336	(6.2)	0.4	(0.3–0.5)

CI: Confidence interval.

Table 2 shows the annual NSI incidence rates for three different hospital sizes in relation to the demographic characteristics of the injured persons, place, timing, and devices involved. Among injured persons 39 years of age or younger, there was a significant trend for the incidence rate to rise as the hospital size increased (p-trend<0.01). Among injured persons in their 40 s and older, there was no significant trend to suggest a relative increase in incidence rates within larger hospitals. When we focused on the injured staff member’s occupation, there was a similar increase in the incidence rate according to larger hospital size. Among the various places the injuries occurred, no hospital size-related trend was observed for emergency departments. NSIs occurred most frequently during the use of medical devices, regardless of hospital size. There was no significant trend indicating a rise in recapping injuries with increasing hospital size.

**Table 2:** Annual incidence rates of needle stick injuries per 100 beds in hospitals of three different sizes.

	399 or fewer beds		400–799 beds		800 or more beds		
	(n = 25 hospitals)		(n = 24 hospitals)		(n = 18 hospitals)		p-value
	Mean	(95%CI)	Mean	(95%CI)	Mean	(95%CI)	(Trend)
Total	4.8	(4.1–5.6)	6.7	(5.9–7.4)	7.6	(6.7–8.5)	<0.01
Ages of injured health care workers (years)							
20–29	2.1	(1.6–2.5)	3.5	(3.0–4.1)	4.5	(3.9–5.0)	<0.01
30–39	1.6	(1.3–1.9)	1.9	(1.6–2.2)	2.2	(1.8–2.5)	<0.01
40–49	0.8	(0.6–1.0)	0.8	(0.7–0.9)	0.6	(0.4–0.7)	0.15
50+	0.4	(0.3–0.4)	0.4	(0.3–0.5)	0.4	(0.3–0.5)	0.2
Occupation of injured health care workers							
Nurses	2.8	(2.5–3.2)	3.3	(2.9–3.8)	3.9	(3.4–4.3)	<0.01
Doctors	1.4	(1.1–1.8)	2.5	(2.1–3.0)	2.6	(2.1–3.1)	<0.01
Place injuries occurred							
Patient room	1.6	(1.3–1.9)	2.0	(1.7–2.3)	2.5	(2.1–2.8)	<0.01
Operating room	1.2	(0.9–1.6)	1.9	(1.6–2.3)	2.0	(1.7–2.3)	<0.01
Outside patient room	0.5	(0.4–0.6)	0.6	(0.5–0.7)	0.8	(0.7–0.9)	<0.01
Outpatient clinic/office	0.4	(0.3–0.5)	0.6	(0.4–0.7)	0.7	(0.6–0.8)	<0.01
Intensive/critical care unit	0.2	(0.1–0.2)	0.2	(0.1–0.3)	0.3	(0.2–0.4)	<0.01
Emergency department	0.2	(0.1–0.3)	0.3	(0.2–0.4)	0.2	(0.2–0.3)	0.2
Timing of injury during device use							
Before use of device	0.2	(0.1–0.3)	0.3	(0.2–0.4)	0.4	(0.3–0.5)	<0.01
During use of device	1.2	(1.0–1.5)	2.1	(1.8–2.4)	1.8	(1.6–2.0)	<0.01
Between steps of a multi-step procedure	0.5	(0.4–0.7)	0.7	(0.5–0.8)	0.9	(0.7–1.1)	<0.01
Disassembling device or equipment	0.2	(0.1–0.3)	0.6	(0.5–0.7)	0.6	(0.4–0.7)	<0.01
In preparation for reuse of a reusable instrument	0.1	(0.1–0.2)	0.2	(0.1–0.2)	0.2	(0.1–0.3)	<0.01
While recapping a used needle	0.6	(0.4–0.7)	0.6	(0.5–0.7)	0.6	(0.5–0.7)	0.76
Withdrawing a needle from rubber or another resistant material	0.1	(0.1–0.2)	0.2	(0.1–0.2)	0.3	(0.1–0.4)	0.01
Other after use-before disposal	0.4	(0.3–0.5)	0.5	(0.4–0.6)	0.8	(0.6–1.0)	<0.01
While putting the device into disposal container	0.4	(0.3–0.6)	0.5	(0.4–0.6)	0.5	(0.5–0.6)	0.03
Restraining patient	0.2	(0.1–0.3)	0.2	(0.1–0.2)	0.3	(0.2–0.4)	<0.01
Devices causing injuries							
Disposable syringe	1.2	(1.0–1.5)	1.7	(1.4–1.9)	1.9	(1.7–2.1)	<0.01
Suture needle	0.7	(0.5–1.0)	1.2	(1.0–1.4)	1.2	(1.1–1.4)	<0.01
Winged steel needle	0.5	(0.3–0.6)	0.8	(0.6–1.0)	0.9	(0.7–1.1)	<0.01
Pre-filled cartridge syringe	0.4	(0.3–0.5)	0.4	(0.3–0.6)	0.7	(0.5–0.8)	<0.01
IV catheter stylet	0.4	(0.3–0.5)	0.4	(0.3–0.5)	0.4	(0.3–0.6)	0.34

CI: Confidence interval.

## Discussion

We determined annual NSI incidence rates in 67 HIV/AIDS referral hospitals in Japan, and clarified incidence trends in hospitals of three sizes, based on the number of occupied beds. The number of NSIs tended to be higher for larger hospital sizes and in workers aged less than 40 years; injury occurrence was more likely to occur in places such as patient rooms and operating rooms. Application of the NSI incidence rates by hospital size, as a benchmark, could allow individual hospitals to compare their NSI incidence rates to those of other institutions, which could facilitate the development of adequate control strategies.

The mean NSI incidence rate in Japan calculated per 100 beds per year was 6.2, which is lower than the corresponding rates in the United States, Taiwan, and South Korea [18]–[20]. The place of injury associated with the highest rate in the United States is the operating room (9.9 per 100 beds), followed by patient room (6.7 per 100 beds) [10]. In contrast, the corresponding figures in this study were 1.7 and 2.0 per 100 beds, respectively, showing relatively fewer injury occurrences compared with the United States. The lower number of NSIs per 100 beds in Japan may be explained by the fact that fewer sharps devices are handled per unit bed, because the mean hospital stay of patients is longer in Japan. The mean hospital stay of patients in 2009 was 18.8 days in Japan, much longer than 4.9 days in the United States [21]. Thus, the number of devices used in Japan per bed on a daily basis, may also be lower, and this could have possibly reduced the overall NSI incidence rates per hospital bed. However, the level of precautions among hospitals may be varied; for example, the studied hospitals might take more precautions since they are conscious of the risks of NSI as they are HIV/AIDS referral hospitals.

Previous studies have shown that NSIs occur more frequently among nurses than doctors [14]–[16]. However, our study suggests there is a chance that doctors are at greater risk of NSIs than nurses. Although, there are (on average) 3.5 times more nurses than doctors within medical institutions in Japan [22], the mean number of NSIs was 1.5-fold higher in nurses than doctors in our study. Since denominator data were lacking in this study (i.e. the number of persons engaged in a particular occupation), precise calculations were not possible. However, previous studies have shown that injuries are more common in surgeons [23] and medical residents [24] in particular, and that the number of NSIs varies among different specialties [25]. It is important to determine, more precisely, the risks for various medical specialists and to provide efficient interventions.

Analysis of NSI incidence rates by age showed that NSI rates in health care workers less than 40 years of age increased with hospital size. In contrast, there was no significant upward trend identified in the NSI rate in personnel over 40 years of age, relative to hospital size. This might be because middle-aged workers at hospitals deal less frequently with blood collection or other tasks that are associated with a high risk of NSI, and are focused on more administrative tasks. It is noteworthy that 51.6% of cases with NSIs were 20–29 years old, while 30.6% were 30–39 years old. Smith et al. reported that nurses 25 years of age and younger at a university affiliated hospital had 2.2 times the risk of NSIs compared with nurses over 25 years old [14]. Young health care workers are clearly a priority when providing educational training concerning NSI prevention [26].

Whether a particular work location carries a high risk of NSIs depends on the types of medical procedures carried out in that place. Procedures with direct access to blood vessels, such as collection of blood samples and placement of intravenous catheters, are frequently undertaken in patient rooms [27], [28]. As a consequence, NSIs occurring in patient rooms account for a high proportion of total NSI incidents [29], [30].

NSIs during recapping might be prevented by the placement of sharps containers in convenient places to help facilitate effective and safe disposal [31]. In this study, NSIs that occurred during recapping accounted for 9.7% of all NSIs, a lower incidence than the corresponding figure in Taiwan (16.5%) [32], but higher than 2006/07 data from the United States (about 4%) [10]. We observed no discernible trend in the number of NSIs due to recapping according to hospital size during this study. The Ministry of Health, Labour and Welfare, Japan has requested prohibiting re-capping in their guideline of infection control [33]. Larger hospitals might take this stance in the prevention from NSIs by prohibiting recapping or installing a needle with automatic needle retraction system resulting in the observed decrease of recapping-related injuries.

The introduction of devices with safety equipment is known to be effective for preventing device-related NSIs [34], [35]. In this study, injuries relating to winged steel needles, IV catheter stylets, and suture needles accounted for 33.9% of incidents; the devices responsible for these injuries were equipped with safety mechanisms, which are available in Japan. However, disposable syringes with safety features have not, to date, been widely used in Japan [36]. Therefore, the introduction of disposable syringes in Japan should be promoted, employing an approach suited to the local health care service.

This study has limitations. An underreporting of injuries may have resulted in an underestimation of true NSI incidence rates. In addition, it should also be noted that staff in larger hospitals may be encouraged to report NSIs and this may account for the higher incidence of NSIs observed in these institutions. Further studies are needed to more accurately estimate the incidence of NSIs. This could be achieved by combining different study designs, such as including an anonymized questionnaire survey. Second, HIV/AIDS referral hospitals are facilities designated by the Ministry of Health, Labour and Welfare. These hospitals must implement a higher than usual standard of occupational infection control and exceed routine levels of NSI control, a potential confounder. Generalizability of our results to other types of hospitals still needs to be considered, especially if the parameters within our work are set as a goal to strive for. Finally, we also need to further elaborate underlying factors, such as number of rooms or patients per staff member and the number of treatments requiring more invasive practices for each institute, in order to validate our findings of the increased incidence rate of NSIs in larger hospitals.

In conclusion, the incidence rate of NSIs tended to be higher for larger hospitals and in workers aged less than 40 years; injury occurrence was more likely to occur in places such as patient rooms and operating rooms. Application of the NSI incidence rates by hospital size, as a benchmark, could allow individual hospitals to compare their NSI incidence rates with those of other institutions, which could facilitate the development of adequate control strategies.
